# Dietary Risk Assessment and Consumer Awareness of Mycotoxins among Household Consumers of Cereals, Nuts and Legumes in North-Central Nigeria

**DOI:** 10.3390/toxins13090635

**Published:** 2021-09-09

**Authors:** Chibundu N. Ezekiel, Kolawole I. Ayeni, Muiz O. Akinyemi, Michael Sulyok, Oluwawapelumi A. Oyedele, Daniel A. Babalola, Isaac M. Ogara, Rudolf Krska

**Affiliations:** 1 Department of Microbiology, Babcock University, Ilishan Remo 121103, Ogun State, Nigeria; kolaay2@gmail.com (K.I.A.); tosin2667@gmail.com (M.O.A.); pelumidele@gmail.com (O.A.O.); 2 Institute of Bioanalytics and Agro-Metabolomics, Department of Agrobiotechnology (IFA-Tulln), University of Natural Resources and Life Sciences Vienna (BOKU), Konrad Lorenz-Str. 20, A-3430 Tulln, Austria; michael.sulyok@boku.ac.at (M.S.); rudolf.krska@boku.ac.at (R.K.); 3 Department of Agriculture and Industrial Technology, Babcock University, Ilishan Remo 121103, Ogun State, Nigeria; akindan15ster@gmail.com; 4 Faculty of Agriculture, Lafia Campus, Nasarawa State University, Keffi 950101, Nasarawa State, Nigeria; isaacjanet2002@yahoo.com; 5 Institute for Global Food Security, School of Biological Sciences, Queen’s University Belfast, University Road, Belfast BT7 1NN, UK

**Keywords:** cereals, consumer awareness, exposure assessment, food safety, harvest, mycotoxins, storage

## Abstract

This study characterized the health risks due to the consumption of mycotoxin-contaminated foods and assessed the consumer awareness level of mycotoxins in households in two north-central Nigerian states during the harvest and storage seasons of 2018. Twenty-six mycotoxins and 121 other microbial and plant metabolites were quantified by LC-MS/MS in 250 samples of cereals, nuts and legumes. Aflatoxins were detected in all food types (cowpea, maize, peanut and sorghum) except in millet. Aflatoxin B_1_ was the most prevalent mycotoxin in peanut (64%) and rice (57%), while fumonisin B_1_ occurred most in maize (93%) and beauvericin in sorghum (71%). The total aflatoxin concentration was highest in peanut (max: 8422 µg/kg; mean: 1281 µg/kg) and rice (max: 955 µg/kg; mean: 94 µg/kg), whereas the totals of the B-type fumonisins and citrinin were highest in maize (max: 68,204 µg/kg; mean: 2988 µg/kg) and sorghum (max: 1335 µg/kg; mean: 186 µg/kg), respectively. Citrinin levels also reached 51,195 µg/kg (mean: 2343 µg/kg) in maize. Aflatoxin and citrinin concentrations in maize were significantly (*p* < 0.05) higher during storage than at harvest. The estimated chronic exposures to aflatoxins, citrinin and fumonisins were high, resulting in as much as 247 new liver cancer cases/year/100,000 population and risks of nephrotoxicity and esophageal cancer, respectively. Children who consumed the foods were the most vulnerable. Mycotoxin co-occurrence was evident, which could increase the health risk of the outcomes. Awareness of mycotoxin issues was generally low among the households.

## 1. Introduction

According to the World Health Organization, an estimated 500 million people, most of whom reside in resource-scarce rural areas of SSA, are exposed to precarious levels of mycotoxins [1]. This worrying statistic could be attributed to a myriad of factors such as climatic conditions that encourage the frequent contamination of food crops by mycotoxigenic fungi, poor agricultural practices, poverty, inadequate knowledge of mycotoxins among primary food producers and a lack of mycotoxin regulations [1,2,3,4]. Mycotoxins of economic and toxicological importance include aflatoxins (AF), citrinin (CIT), deoxynivalenol (DON), fumonisins (FUM), ochratoxin A (OTA) and zearalenone (ZEN) [3]. However, improvements in analytical techniques have led to an upsurge of research interest in “emerging” mycotoxins such as beauvericin (BEAU), enniatins (ENN) and moniliformin (MON), which are now frequently found in various foods worldwide [5,6]. Depending on the concentration and type of mycotoxins ingested, human exposure could result in serious health effects ranging from acute (e.g., headache and vomiting) to chronic (e.g., cancers, stunting, hepatomegaly, suppression of immunity) and, in the worst cases, deaths [3,7].

Similar to other regions in SSA, mycotoxin contamination of foods at unsafe levels is commonplace in Nigeria [8,9,10,11,12,13,14,15], with most reports covering foods consumed in the north-central region [9,10,11,12,13,16,17]. In one of the recent studies, more than 45% of grain-based uncooked flour (mostly from maize, rice and sorghum) contained at least two mycotoxins, including the carcinogenic AFB_1_ and FB_1_. The maximum concentrations of AFB_1_ and FB_1_ in the staples were 37.3 µg/kg and 7387 µg/kg, respectively [10]. Obviously, mycotoxin contamination of foods could threaten the actualization of goal number 3 (good health and wellbeing) of the United Nations Sustainable Development Goals in this region.

Irrespective of the available data on the occurrence of mycotoxins in foods from north-central Nigeria, there is no comprehensive study that elucidates the spectra of mycotoxins and other toxic metabolites in household foods across the harvest and storage seasons. Seasonal variations as well as harvest and storage practices can influence mycotoxin levels in food [8,18]. Furthermore, there is sparse documented evidence on the level of mycotoxin awareness and the food handling practices that influence food contamination by mycotoxins in north-central Nigeria. Thus, in view of the need to provide robust surveillance data to drive urgent interventions towards ensuring safe foods and safeguarding consumer health, the present study was designed. This study profiled mycotoxins in foods consumed by several households in Nasarawa and Niger states during the harvest and storage seasons, estimated the corresponding risks of mycotoxin exposure among the consumers and assessed the consumer awareness levels of mycotoxins as well as the food handling practices adopted by households in the two states.

## 2. Results and Discussion

### 2.1. Distribution of Multi-Mycotoxins in Foods

Twenty-six major mycotoxins and their metabolites (Table 1) and 121 other fungal, bacterial, algal and plant metabolites (Tables S1 and S2) were detected in the food samples. The mycotoxins included AF, FUM, CIT, NIV, OTA, STER, ZEN and the emerging *Fusarium* mycotoxins BEAU and MON. A similar diversity of mycotoxins and other metabolites was previously reported in cowpea [19], maize [8,13,15], peanut [14] and rice [20] in Nigeria and elsewhere in sub-Saharan Africa [21,22,23,24,25,26], emphasizing that mycotoxin contamination of foods is an important food safety challenge that is yet to be solved in sub-Saharan Africa. To date, however, only few mycotoxins including AFs, FUM, OTA and ZEN determined by ELISA or thin-layer chromatography were reported in sorghum and millet in Nigeria [17,27]. Consequently, the present paper reports, for the first time, LC-MS/MS-based surveillance data of multiple mycotoxin contamination in sorghum in Nigeria.

#### 2.1.1. Aflatoxins

Aflatoxins were found in all the food types analyzed except in the sample of millet. AFB_1_ was the most prevalent AF type in the foods, occurring in 64% of peanut, 66% of maize, 57% of rice and 46% of sorghum samples at mean (max) concentrations of 939 (6413) µg/kg, 99.4 (2277) µg/kg, 44 (416) µg/kg and 19.4 (138) µg/kg, respectively (Table 1). The mean concentration of AFB_1_ in the peanut samples was 8 and 20 times higher than the mean values (117.8 µg/kg and 47 µg/kg) previously reported in peanuts from Nigeria [14] and Cameroon [21], respectively. The mean level of AFB_1_ found in maize in the present study was, however, lower than the value of 394 µg/kg previously reported in stored maize from different agro-ecological zones of Nigeria [8] but was 2–8 times higher than the mean concentrations (39 µg/kg and 11.8 µg/kg) reported in maize from Togo [24] and China [28], respectively. Similarly, AFB_1_ in rice was lower than the level of 60.3 µg/kg previously reported in rice in Nigeria [11], but 7 times higher than the concentration of 5.84 µg/kg reported in rice from Pakistan [29]. In addition, the analyzed sorghum samples contained lower mean AFB_1_ levels than the concentration of 29.5 µg/kg reported in Ethiopia [22], but the level was 8–16 times higher than the values of 2.32 µg/kg and 1.24 µg/kg reported in sorghum from Egypt and Tunisia, respectively [30].

AFG_1_ was also quantified in the food samples, albeit at lower incidences (17–33%) and concentrations (3–9 times lower mean levels compared to AFB_1_) except for rice, where the AFG_1_ mean concentration (145 µg/kg) was unusually high and as much as 3 times higher compared to AFB_1_ (44 µg/kg). This unusual trend suggests that AFG_1_ producing strains such as *Aspergillus austwickii*, *A. cerealis* and *A. aflatoxiformans* [31] contaminated the rice samples during the pre- and/or post-harvest stages. These AFG_1_ producers were, however, not found in the rice samples, but were mostly isolated from the soil environment where the rice samples were cultivated [32]. Other AF types quantified were AFB_2_, AFG_2_, AFM_1_, AFM_2_ and AFP_1_. AFB_2_ and AFG_2_ were both found in all foods where their parent toxins occurred, while AFM_1_ was found in 42% of peanut (mean: 80.8 µg/kg), 25% of maize (mean: 10.4 µg/kg), 9% of rice (mean: 14.2 µg/kg) and 17% of sorghum (mean: 1.60 µg/kg). AFM_2_ was found only in maize, while AFP_1_ was in maize and sorghum. These other AF types have previously been reported in cereals and nuts in Nigeria [8,13,14]. Summation of aflatoxins B_1_, B_2_, G_1_ and G_2_ (referred to as AF total) occurred in 64% of peanut (max: 8422 µg/kg; mean: 1281 µg/kg), 67% of maize (max: 3863 µg/kg; mean: 128 µg/kg), 57% of rice (max: 955 µg/kg; mean: 93.9 µg/kg) and 46% of sorghum (max: 158 µg/kg; mean: 23.1 µg/kg). Overall, AF was predominant in maize compared to other food types, whereas higher maximum and mean concentrations were reported in peanut, in accordance with the report of Abia et al. [21] but in disagreement with Warth et al. [25] and Ediage et al. [23], who found higher AF concentrations in maize compared to peanuts in Burkina Faso, Cameroon and Mozambique. In addition, out of the seven cowpea samples analyzed in this study, only one sample was contaminated with mycotoxins including AFB_1_ (122 µg/kg), AF total (133 µg/kg), STER (0.49 µg/kg) and CPA (476 µg/kg). The low contamination of cowpea by AF agrees with reports of Afolabi et al. [19] and Maringe et al. [33], who found AF in less than 10% of cowpea from Nigeria and Zimbabwe, respectively.

#### 2.1.2. Fumonisins

Fumonisins were quantified only in the cereals (maize, rice and sorghum) (Table 1). Precisely, fumonisin B_1_, a class 2B carcinogen [34], contaminated 93% of maize (max: 47,168 µg/kg; mean: 2078 µg/kg), 22% of rice (max: 413 µg/kg; mean: 244 µg/kg) and 50% of sorghum (max: 475 µg/kg; mean: 36.7 µg/kg). The sum of fumonisins B_1_, B_2_, B_3_ and B_4_ was found in 93% of maize (max: 68,204 µg/kg; mean: 2988 µg/kg), 26% of rice (max: 601 µg/kg; mean: 303 µg/kg) and 54% of sorghum (max: 674 µg/kg; mean: 203 µg/kg). The mean level of FB_1_ in maize was four times higher than the levels of 508 µg/kg and 504.8 µg/kg reported as mean FB_1_ concentrations in maize from Cameroon [21] and Togo [24], respectively. Similarly, the mean concentration in the present study was higher than the mean concentration reported for maize from the 2009 season in Cameroon (1329 µg/kg [23]) and for maize from five agro-ecological zones of Nigeria (1552 µg/kg [8]), but was similar to the mean level in maize from the 2010–2011 season in Cameroon (2072 µg/kg [23]). However, the findings presented here contradict the reports of a recent study from a south-eastern state (Anambra) in Nigeria, where fumonisins were not detected in 36 maize samples [15]. Obvious reasons for this disparity could be geographical location and sampling techniques. All the households included in this present study are located within the derived savannah and southern Guinea savanna agro-ecological zones, where FB_1_ contamination of maize has been shown to be more prevalent [8,13].

The mean concentration of FB_1_ in rice was as much as 11 and 13 times higher than the concentrations (22.9 µg/kg and 18.5 µg/kg) previously reported for rice in Nigeria [20] and Pakistan [29], respectively. In addition, the mean level of FB_1_ quantified in the sorghum samples was about 10 times higher than the value of 14.2 µg/kg reported for sorghum from Ethiopia [22]. So far, it is evident that rice and sorghum are less susceptible to fumonisins than maize. Fumonisins were not detected in peanut, which agrees with previous reports from Nigeria [14] and Sierra Leone [35] but contradicts the findings from Cameroon, where FB_1_ was found in peanut, albeit at very low mean concentration of 5 µg/kg [21].

#### 2.1.3. Other Mycotoxins

OTA was detected in less than 15% of each food type, with the highest concentration in maize (94 µg/kg), while CIT contaminated 4%, 30%, 42% and 57% of peanut (max: 15 µg/kg; mean: 9.8 µg/kg), rice (max: 358 µg/kg; mean: 121 µg/kg), sorghum (max: 1335 µg/kg; mean: 186 µg/kg) and maize (max: 51,195 µg/kg; mean: 2343 µg/kg) samples, respectively (Table 1). Dihydrocitrinone (DHC), a citrinin metabolite, was detected only in maize, rice and sorghum, with the highest mean concentration (70.4 µg/kg) in sorghum. OTA, a nephrotoxin, has been less frequently detected, and at relatively low concentrations, in diverse foods in Nigeria including maize, peanut and rice [8,13,14,20]. However, another nephrotoxin [36], citrinin, and its metabolite DHC, were more prevalent in the cereals than the nut samples, suggesting that CIT is a major contaminant in cereals. Of all the cereals, maize contained the highest mean CIT concentration, at a level 13 and 19 times higher than rice and sorghum, respectively. The mean CIT and DHC concentrations were lower than the respective values (16,773 µg/kg and 1820 µg/kg) previously reported in maize for *ogi* fermentation from Nigeria [37]. Similarly, the mean CIT level in the present study was lower than the mean concentration (4586 µg/kg) reported in ear-rot infested maize in Nigeria, but was much higher than the levels reported in maize from Burkina Faso (1784 µg/kg [25]), Cameroon (33 µg/kg [21]) and Mozambique (545 µg/kg [25]). ZEN was quantified in peanut, maize, rice and sorghum, with the highest occurrence (57%) and concentration (max: 8.7 µg/kg; mean: 2.8 µg/kg) in rice.

STER contaminated 48%, 40%, 26% and 13% of the rice, peanut, maize and sorghum samples, respectively, at concentrations reaching 30 µg/kg in peanut, while cyclopiazonic acid (CPA) and NIV were quantified only in peanut (mean: 2345 µg/kg; mean: 32 µg/kg, respectively) and maize (mean: 701 µg/kg; mean: 20 µg/kg) (Table 1). CPA was previously reported at lower concentrations in peanut from Nigeria (65.8 µg/kg [14]) and Mozambique (763 µg/kg [25]). The millet sample analyzed in this study contained only DON (49 µg/kg) and MON (282 µg/kg). Other toxicological important mycotoxins quantified at high frequencies in at least two of the cereals and nuts include BEAU and MON.

### 2.2. Seasonal Distribution of Multi-Mycotoxins in Foods

Seasonal variations were observed in the mycotoxin contents of the foods consumed by households in the two north-central Nigerian states during the harvest and storage seasons (Table 2). With the exception of maize and rice, the mean concentrations of aflatoxins in the cereals and nuts were higher at harvest than in the storage season, with statistically significant (*p* < 0.05) higher mean concentration reported only in peanut (AFB_1_: 1110 µg/kg at harvest vs. 462 µg/kg in storage; AF total: 1457 µg/kg at harvest vs. 630 µg/kg in storage). The higher incidences and mean concentrations of aflatoxins in peanut during harvest when compared to the storage season may be due to the shelling and sorting steps applied to the peanuts between the time of harvest sampling and storage. During the harvest season, ~90% of the peanuts sampled from the households were unshelled; as such, bad kernels (possibly containing high aflatoxin levels) were not sorted out. Conversely, during the storage season, the peanut samples collected were already shelled and carefully sorted before storage. Postharvest practices such as shelling and sorting have been reported to reduce the aflatoxin contamination of groundnut and its products [38,39]. The aflatoxin level in maize was significantly (*p* < 0.05) higher during storage (AFB_1_: 167 µg/kg; AF total: 236 µg/kg) than at harvest (AFB_1_: 61 µg/kg; AF total: 68 µg/kg) (Table 2). Unlike the groundnut samples, maize and rice had higher aflatoxin contents during the storage season, agreeing with previous reports on the higher aflatoxin levels in stored grains compared to freshly harvested grains [40,41,42]. Some obvious reasons for the recorded data were poor drying and storage practices adopted by the households. Keen observations of the practices at the various communities revealed that households dried the foods on bare ground or at best on elevated surfaces in outdoor environments, where these foods were exposed to unstable weather conditions. To complicate the situation, the households stored their maize in non-hermetic (e.g., jute and woven polypropylene) bags. These bags are poorly constructed because they allow for the passage of air, which expose the stored foods to fungal spores [43]. Poor storage practices have been shown to contribute to increased aflatoxin contamination in maize [41,42,44,45].

Fumonisins were quantified at significantly (*p* < 0.05) higher levels in maize during harvest (FB_1_: 2462 µg/kg; sum of FB_1_, B_2_, B_3_ and B_4_: 3554 µg/kg) than in storage (FB_1_: 1487 µg/kg in storage; sum of FB_1_, B_2_, B_3_ and B_4_: 2117 µg/kg in storage) (Table 2). The observed marked difference in fumonisin levels in maize in both seasons agrees with previous reports that showed higher fumonisin levels in maize and cereal-based foods at harvest compared to storage [18,46,47,48,49]. The reduction in FUM levels in the maize samples between harvest and storage may have been due to the biochemical reactions, mediated by pests and enzymes, for the conversion of parent FUM to masked forms [50,51]. The variations in the mean concentrations of FB_1_ and the sum of the fumonisins in rice in both seasons were minimal and statistically insignificant. Similarly, the mean concentrations of other *Fusarium* mycotoxins (NIV, MON and ZEN) in the cereals and nuts did not vary significantly in both seasons. On the other hand, significantly (*p* < 0.05) higher mean concentrations of CIT and its metabolite DHC were respectively quantified in maize during storage (4331 µg/kg and 96 µg/kg) compared to maize at harvest (305 µg/kg and 21 µg/kg) (Table 2). Similarly, OTA levels were generally higher in the foods during the storage season than at harvest, whereas CPA levels were higher in groundnut and maize during harvest than in the storage season. CPA levels are known to be reduced in the presence of oxygen [52,53]; therefore, it is likely that oxygen diffused into the porous storage bags used by the households, causing the degradation of CPA during the storage season. The variations of mycotoxins in sorghum during both seasons could not be compared due to the limited number of samples collected during the harvest season. To the best of our knowledge, this is the first comprehensive report on multiple mycotoxin comparisons in household foods during harvest and storage seasons in Nigeria, and specifically, the first seasonal comparative report of CIT in maize globally.

### 2.3. Occurrence of Major Mycotoxins in Foods in Two North-Central Nigerian States

The occurrences of major mycotoxins in grains from Nasarawa and Niger states are presented in Table 3. Generally, the levels (maximum and mean) of mycotoxins were higher in the foods from Nasarawa state than in foods from Niger state. For example, as much as three times higher and significant (*p* < 0.05) mean concentrations of AFB_1_ and AF total were quantified in foods from Nasarawa state (326 µg/kg and 452 µg/kg, respectively) compared to Niger state (118 µg/kg and 129 µg/kg, respectively). Similarly, the respective mean concentrations of CIT, FB_1_ and the sum of fumonisins were significantly (*p* < 0.05) higher in Nasarawa state (2104 µg/kg, 2041 µg/kg and 2883 µg/kg) compared to Niger state (1487 µg/kg, 1552 µg/kg and 2264 µg/kg). The mean CPA level in the foods was about four times higher in Nasarawa state (1572 µg/kg) than in Niger state (436 µg/kg).

The observed higher incidence and mean mycotoxin concentrations in foods from Nasarawa compared to Niger state suggest that foods in this state are more prone to mycotoxin contamination, and consumer populations may be at a higher risk of mycotoxicosis. Several possible combinations of factors may be responsible for this observation, and they include geography and rainfall patterns, poor food handling (drying and storage) practices, fungal diversity and distribution and the awareness level of the households, among other factors [2,8,43,54]. Higher aflatoxin contents have previously been reported in maize from Nasarawa (derived savannah zone) than in Niger (southern Guinea savannah zone) [8]. The rainfall pattern is higher (1300–1500 mm) in the derived savannah than in the southern Guinea savannah region (1000 and 1300 mm) [8,54]. In addition, some of the visited communities in Nasarawa were situated about 20 km away from any visible major road and infrastructure that could attract development and community enlightenment. This will obviously play a role in limiting the accessibility of community developers and the knowledge of households on good food handling and storage practices usually disseminated by extension workers. Furthermore, it was observed that a higher diversity of fungi, including the toxigenic species, were found in the foods and agricultural soils from Nasarawa compared to Niger state [32]. Overall, the mean concentrations of the carcinogenic AFB_1_, FB_1_ and the nephrotoxic CIT were very high in the foods irrespective of the location, suggesting a possible health risk to consumers in these states and to wherever these foods are transported.

### 2.4. Levels of Regulated Mycotoxins Exceeding Legislated Thresholds in the Foods

The proportions of food samples exceeding the levels stipulated by the European Union as well as the food regulatory agency in Nigeria are presented in Table 4. The levels of AFB_1_, AF total, the sum of FB_1_ and FB_2_ and OTA exceeded stipulated EU limits in various food types. About 51%, 44%, 22%, 21% and 14% of peanut, maize, rice, sorghum and cowpea samples contained a total of AF at concentrations above the EU threshold of 4 µg/kg, while the same proportions of samples, except for maize (35%) and rice (13%), exceeded the maximum acceptable limit of 10 µg/kg of total AF adopted in Nigeria. Overall, the total levels of AFB_1_ and AF in all the foods were up to 470 and 320 times higher than the EU regulatory limits of 2 µg/kg and 4 µg/kg, respectively. As much as 42% of the maize samples contained a sum of FB_1_ and FB_2_ above the EU threshold of 1000 µg/kg. About 2%, 4% and 8% of maize, rice and sorghum samples, respectively, contained OTA at levels exceeding the 5 µg/kg set by EU. When all foods were taken into consideration, 40%, 24% and 2% of the foods contained AF total, sum of FB_1_ and FB_2_ and OTA at levels exceeding the EU’s maximum acceptable limits (Table 4). The economic impacts of mycotoxin contamination of foods on households and the country could be troubling [1,55]. In most cases, rural households—mostly farmers—supply food crops to local markets where aggregators can buy the foods and sell them off to the international market. The exceedance of at least one-third and 42% of all the foods and maize for the EU threshold of 4 µg/kg of total aflatoxin and 1000 µg/kg of total fumonisins, respectively [56], is worthy of note. In the case that these foods find their way into the international market, the consignments will not pass the stringent measures put in place by the European Union. Consequently, the food crops will be confiscated and destroyed, and economic sanctions may be imposed on the country, thus leading to economic loss and poverty at the household level.

### 2.5. Estimated Dietary Exposures and Health Risks in Average Consumers of the Foods

The chronic mycotoxin exposures from the consumption of the foods (cereals and nuts), HBGVs and reference points applied in the mycotoxin risk assessments as well as the calculated health risk values are presented in Table 5. The trend of mycotoxin exposures for all the food types was children > adolescents > adults. Similarly, the calculated MOEs for all mycotoxins, except FUM, and liver cancer risk values for AFB_1_ were highest in the population of children than in the adolescent and adults. Specifically, the average calculated PDIs for children were about two times and six times higher than the average PDIs estimated for adolescents and adults, respectively. For example, the average AFB_1_ and AF total exposures/PDIs for children who consumed peanut (4883 ng/kg bw/day and 6661 ng/kg bw/day) were more than two times higher than the corresponding exposures in adolescents (1953 ng/kg bw/day and 2664 ng/kg bw/day) and six times higher than the exposures in adults (803 ng/kg bw/day and 1096 ng/kg bw/day, respectively). The observed trend agrees with previous reports that suggested higher exposures and risk values in populations of infants and children compared to adolescents and adults [14,57,58].

With respect to food type, as much as 8–98 times higher average PDIs of the population categories (children, adolescents and adults) were calculated for consumers of aflatoxin-contaminated peanut than for the consumers of other foods (Table 5). The exposure levels to total AF estimated in the present study for peanut consumers was as much as six times higher than the levels reported in a nationwide study in Nigeria (187–1124 ng/kg bw/day) [14], whereas the exposure levels for maize consumers were similar to the values obtained in Ondo state, Nigeria (138–830 ng/kg bw/day) [58] but lower than previous reports from a nationwide study in Nigeria (318–1909 ng/kg bw/day) [57] and in Somalia (649–1614 ng/kg bw/day) [26]. Consequent to the estimated high exposures, the MOEs were far lower than 10,000, with the values for peanut consumers being the lowest (0.026–0.16) compared to other foods: maize (0.20–1.23), rice (0.41–2.50) and sorghum (2.51–15.3). Based on the calculated MOEs, a risk of aflatoxicosis was indicated for all the population, irrespective of the food consumed. An MOE less than 10,000 for a genotoxic and carcinogenic substance based on the BMDL_10_ is considered to represent a risk to public health [59]. Thus, in all cases (population and food type) in this present study, there are indications of chronic health risks triggered from exposure to aflatoxins in all the populations in the studied states and wherever else the foods are disseminated. The liver cancer risk due to the consumption of AFB_1_-contaminated peanut was highest (41–247 cancers/year/100,000 population) compared with cancer from the consumption of other food types: maize (6–33 cancers/year/100,000 population), rice (2–10 cancers/year/100,000 population) and sorghum (1–3 cancers/year/100,000 population) (Table 5). Exposure to AFB_1_ can cause human hepatocellular carcinoma, and this can be aggravated by simultaneous exposure to HBV [3,60]. Of all the food types, the consumption of groundnut constituted the highest health risk; however, children who consumed all the foods were the most at risk due to the lowest recorded MOE. In view of the high incidence (13.6%) of HBV infection in Nigeria [61] and the calculated HCC cases due to the consumption of aflatoxin-contaminated foods in this study, the principle of “as low as reasonably achievable (ALARA)” advised by JECFA and the EFSA should be enforced by food risk managers to protect consumers of these foods [62]).

For all other mycotoxins (BEAU, CIT, FUM and MON), the average PDIs were higher for maize consumers compared to consumers of other foods (Table 5). For example, the average CIT exposures in the populations who consumed maize (2.5–15.4 µg/kg bw/day) were 28 times higher than the exposures due to rice (0.08–0.53 µg/kg bw/day) and sorghum (0.09–0.55 µg/kg bw/day) consumption. CIT exposure levels for maize consumers exceeded the 0.2 μg/kg bw per day level of no concern for nephrotoxicity [36], suggesting possible health risks to the maize consumers. This is supported by the calculated MOE values, which were far less than 10,000 and in the range of 0.013–0.08 in the maize-consuming population. CIT is considered to be both a nephrotoxin [76,77] and genotoxin [78,79]. Similarly, the average FUM exposures as a result of maize consumption (3.24–19.7 µg/kg bw/day) were 15 and 33 times higher than the exposures from rice (0.22–1.34 µg/kg bw/day) and sorghum (0.10–0.60 µg/kg bw/day) consumption, respectively. The FUM exposure for children who consumed maize was higher than the APDI for fumonisins (14.14 μg/kg bw/day) calculated for children in South Africa, where the esophageal cancer rate was reported to be high [80,81]. Overall, the estimated FUM exposure levels for maize consumers in the present study exceeded the 2 μg/kg bw per day group TDI for the sum of fumonisins [75], which indicates that the maize consumers may be at risk of several health outcomes. Human exposure to dietary fumonisins has been linked to esophageal cancer [82], neural tube defects [83] and the impairment of growth in children [84,85,86]. Although the incidence of esophageal cancer is relatively low in Nigeria [87], continuous consumption of highly contaminated maize could substantially increase the risk of esophageal cancer development in view of the vulnerability and low detoxification capacity of children [88].

An important aspect to consider is the possible toxicological interactions between the various mycotoxins quantified in the foods. Co-exposures to AF and FUM have been suggested to induce chronic health effects such as stunting in children [3,85], whilst the co-occurrence of CIT with OTA observed in about 12% of the cereals could lead to synergistic interactions with possible health risk outcomes [89,90]. The co-occurrence of CIT with AFB_1_ and FB_1_, in at least 30% of the cereal food types, further raises concerns for consumer health; however, the interactive effects of these combinations in humans are not yet completely understood. BEAU, which was prevalent in the foods, can interact with OTA in vitro to induce DNA damage [91], while MON exhibits acute toxicity in rats [92]. In view of the recorded food contamination data, the calculated exposure risks and possible combinatory effects of the mycotoxins found in the present study, urgent and defined measures of risk management in the populations are required.

### 2.6. Consumers’ Characteristics, Grain Handling and Awareness of Mycotoxins in Foods

#### 2.6.1. Consumers’ Characteristics and Grain Handling

The survey data for respondents’ personal factors and grain handling parameters by location are presented in Table 6. The mean ages of respondents in the sampled states are comparable, although the respondents from Niger state (36.9 ± 16.6) were slightly older than those from Nasarawa state (33.9 ± 13.8). Generally, the age distribution shows that most of the respondents were relatively young, and as farmers, they were still in their active age. Thus, food safety and health-related information are *sine qua non* to their productivity [2,93]. With more than 50% of the respondents from both states having at least secondary education, it is expected based on past studies that these respondents should have relevant and adequate information relating to food safety as producers and consumers of the foods [2,93,94]. In addition, the results indicate a large participation of women in farming and food processing. At least 42% and 51% of the farmers were females in Nasarawa and Niger states, respectively. This shows the significant contribution of women to food security in the studied states in Nigeria.

A high proportion of farmers (95% and 99%) in both states (Nasarawa and Niger states, respectively) plant local seeds because they do not have access to genetically improved and certified seeds. Consequently, these farmers (Nasarawa: 81%; Niger: 94%) source their seeds from the previous harvest (Table 6). Thus, there is a need for these farmers to explore improved and effective grain storage practices to keep the best-quality seeds in circulation throughout the seasons [43]. The pre-storage grain handling practices commonly employed by farmers in the study areas include drying (Nasarawa: 99%; Niger: 89%), shelling (Nasarawa: 26%; Niger: 41%) and packaging (Nasarawa: 72%; Niger: 48%). The majority (Nasarawa: 90%; Niger: 74%) of households stored grains in bags for more than 3 months (Nasarawa: 77%; Niger: 78%). More than one half (Nasarawa: 59%; Niger: 70%) of the households protected their stored grains from rain/water, and in the event of grain exposure to rain, 71% and 91% of these respondents in both states, respectively, adopted sun-drying as a coping strategy for the soiled grains (Table 6). Protection of grains from rain/water during storage is critical to keep stored grains safe and the mycotoxin levels minimal [43,95,96].

The simple traditional grain processing methods predominantly adopted by the respondents/households include sorting (Nasarawa: 90%; Niger: 95%) and winnowing (Nasarawa: 79%; Niger: 63%). The washing of grains in cold water or hot water and fermentation were also adopted by less than one half of the responding households in Nasarawa (34%, 44% and 45%, respectively) and Niger (14%, 21% and 23%, respectively) (Table 6). Grain washing (especially in cold water), sorting and winnowing have been reported to lower mycotoxin levels of grains due to the removal of damaged (broken, discolored and insect-infested) grains [97,98,99,100]. Although grain processing by fermentation is moderately adopted in both study areas, fermentation leads to significant mycotoxin reduction during the processing of cereal-based grains into fermented foods [101,102,103,104,105].

#### 2.6.2. Mycotoxin Awareness among Grain Consumer Households

Data on the awareness of households consuming grains of mycotoxin-related topics are given in Table 6 and Table 7. At least 80% of the respondents in each state (Nasarawa: 84%; Niger: 80%) indicated that they could identify molds in foods and stored grains by the discolorations on the food items. However, only 43% and 15% of these respondents from Nasarawa and Niger states, respectively, had ever heard of or knew what mycotoxins were, while only 26% and 11%, respectively, were aware of possible food handling practices to reduce mycotoxins in food (Table 6). The higher awareness level in Nasarawa may be due to the higher level of education among respondents in the state compared to Niger state. The predominant food handling practice identified to reduce mycotoxin contamination of grains was the early harvesting of grains; this was adopted by 10% and 75% of the respondent households in Nasarawa and Niger, respectively. Early harvesting has been reported to prevent field-to-store pests and pathogen infestation of grains, thereby limiting mycotoxin contamination [106]. This practice may have significantly impacted the vulnerability of the grains to mycotoxins as well as lowering the mycotoxin levels recorded in foods from Niger state compared to foods from Nasarawa state. A community campaign was reported to be the major source of respondents’ awareness (46% positive responses) to mycotoxins in both states; this reflects the efforts of previous food safety and mycotoxin-related projects in both states [9,10,11,27,32]. Other sources of mycotoxin awareness in Nasarawa and Niger states include extension education offered by agricultural and public health officers (29% vs. 36%, respectively) and mass media (25% vs. 18%, respectively) (Table 6).

In order to determine the significance of the factors influencing mycotoxin awareness among the respondents, the Logit regression analysis was performed, and the data are presented in Table 7. The chi-square values (Nasarawa: 25.0; Niger: 93.0; pooled: 61.5) for the hypothesized determinants of respondents’ awareness of mycotoxins were statistically significant (*p* < 0.05). This indicates that the model is significant and of good fit for the analysis. The Nagelkerke’s R^2^ values are greater than 0.5, corroborating the chi-square data for the fitness of the model (above 50%). Overall, factors increasing the probability of awareness of mycotoxins among the farmers include education level and their exposure to food handling practices (*p* ≤ 0.05). Thus, this result is consistent with previous studies [2,107].

## 3. Conclusions

Herein, a comprehensive overview of the multiple mycotoxin contamination of diverse foods including cereals, nuts and legumes consumed by households in two north-central Nigerian states is reported. High levels of AF, CIT and FUM in addition to other mycotoxins were quantified in all the foods, resulting in significant calculated exposure risks, especially to cancers. The calculated risks associated with the consumption of the mycotoxin-contaminated foods were higher in the population of children, suggesting severe negative health impacts into adulthood following chronic exposure to the food toxins. In addition, the storage of maize under household traditional conditions for three months elevated AF and CIT concentrations. The awareness of the food producing and consuming households on mycotoxin issues was generally low. Consequently, interventions aimed at limiting mycotoxins in foods from harvest to storage should be prioritized. Based on the evidence of the data available in this study, the regulation of CIT and FUM in cereals should be considered in Nigeria. In the meantime, the ALARA principle is suggested in addition to the routine surveillance of foods. Furthermore, more efforts should be directed towards awareness as well as educational intervention in the rural communities where food production dominates. These recommendations are crucial to improve the human capacity to produce and process safer foods for a healthier nation.

## 4. Materials and Methods

### 4.1. Food Sampling

A longitudinal household survey was conducted in two states (Nasarawa and Niger) in north-central Nigeria to sample foods at harvest and storage in order to determine the variations in mycotoxin contamination across the seasons. In each state, food samples were collected within three randomly selected communities where the households were fully subsistence farmers. A total of 250 food samples comprising of cereals (maize (*n* = 142), millet (*n* = 1), rice (*n* = 23) and sorghum (*n* = 24)), legumes (cowpea (*n* = 7)) and nuts (peanut (*n* = 53)) were collected from two states (Nasarawa (*n* = 170) and Niger (*n* = 80)). Harvest samples were collected in September 2018, whilst storage sampling was conducted in January 2019. Precisely, 143 and 107 food samples were collected during the harvest and storage seasons, respectively. Harvest food samples were collected from randomly selected households within one week after the crops were harvested, while storage samples were collected from stores of the same households three months after food storage.

Each food sample (~1 kg) was taken from different parts of the food lot gathered at harvest or the stored grains in the storage structures during the storage season. All food samples were collected into polyethylene bags and transported to the laboratory. Thereafter, the samples were comminuted immediately in an electric blender (MX-AC400, Panasonic, India) prior to storage at −20 °C for further analysis.

### 4.2. Questionnaire Administration to Households

In order to elicit information related to grain handling and storage practices as well as the knowledge on mycotoxin issues and the factors influencing mycotoxin awareness among the households, revised validated structured questionnaires [9,21] were administered at the point of food sampling. A total of 155 questionnaires (Nasarawa: 82; Niger: 73) were administered to the head of the households in local languages after translation.

### 4.3. Determination of Multi-Mycotoxins in Food

The spectrum and levels of more than 500 compounds, including mycotoxins, plant and other microbial metabolites, were detected and quantified in the foods by a dilute and shoot LC–MS/MS method [108]. All chemicals, reagents and mycotoxin standards used for LC-MS/MS analysis were obtained as previously reported in Abia et al. [109]. Five grams of a food sample was weighed into a 50 mL polypropylene tube (Sarstedt, Nümbrecht, Germany) and 20 mL of extraction solvent (acetonitrile/water/acetic acid 79:20:1, *v*/*v*/*v*) was added. Details of the apparent recovery of the method determination were as given in Abia et al. [109], while extract dilution and injection into the LC–MS/MS instrument were performed according to Sulyok et al. [110]. The LC-MS/MS system and chromatographic separation details were as detailed in Abia et al. [109], whereas the chromatographic method, chromatographic and mass spectrometric parameters were as described by Sulyok et al. [108]. The MRM detection window of each analyte was set to its expected retention time ±20 s and ±26 s in the positive and the negative modes, respectively. The confirmation of identified positive analytes was as previously detailed in European Commission decision 2002/657 [111], Sulyok et al. [108] and Sulyok et al. [110]. A verification of the accuracy of the method for food analysis was performed by participation in inter-laboratory comparison studies organized by BIPEA (Gennevilliers, France). At present, 94% of the more than 1100 results submitted for different types of grains and nuts were in the satisfactory range of z-scores between −2 and 2. An expanded measurement uncertainty of 50% was proposed based on the related data [112]. The limits of detection and limits of quantification of the metabolites were determined based on the standard deviation of the samples spiked at low concentration levels following the EURACHEM guide [113].

### 4.4. Estimation of Dietary Mycotoxin Exposure and Risk Assessment

#### 4.4.1. Point Estimates of Exposure

Chronic mycotoxin exposure among households who consumed the sampled foods daily in Nigeria was estimated by the deterministic approach involving the Average Probable Daily Intake (APDI) method [63,64]. The APDI (ng/kg bw/day) was estimated by multiplying the mean mycotoxin contamination levels (ng/g) in a specific food with the estimated average consumption of the specified food (g/person/day). The product was then divided by the average body weight (kg) of individuals in the population (children, adolescents and adults). Food consumption data (g/person/day) for the foods, except peanut, were estimated according to the consumption data in the NBS/World Bank [66] Living Standards Measurement Study (LSMS)—Panel (Wave 3), and Claro et al. [67] Adult Equivalent Conversion Factor based on WHO Recommended Dietary Allowances according to age and gender. Food consumption data (g/person/day) for peanut were estimated according to WHO [65]. Average body weights were assumed as 10 kg for children and 25 kg for adolescents [68,69,70], while a population-based average body weight of 60.8 kg was estimated for adults from questionnaire data in this study. The dietary exposures were estimated for only the major mycotoxins of public health significance (AFB_1_, total AFs, CIT and FB_1_ + FB_2_ + FB_3_ + FB_4_) and the emerging mycotoxins (BEAU and MON; [6]).

The handling of left-censored mycotoxin contamination data (that is, data below LOD) is critical in exposure and risk assessment estimations; thus, we considered these data according to the guidelines of IPCS/GEMS [114]. For most mycotoxins, more than 60% of the food samples had contamination levels above the LOD. In cases in which less than 60% of samples of specific food types contained levels below the LOD and left-censored data were reported, the exposure of the food consumer was estimated by substituting LOD/2 (the middle bound) for <LOD in order to obtain an appropriate exposure estimate [62]. Due to the small sample sizes for cowpea (*n* = 7) and millet (*n* = 1) and their very low mycotoxin contamination data, these two foods were not included in exposure and risk assessment calculations.

#### 4.4.2. Risk Characterization of Dietary Exposures by Margin of Exposure and Health-Based Guidance Value Approaches

The margin of exposure (MOE) approach recommended by EFSA [71] and JECFA [72] was applied to assess the risks from the household consumption of foods ladens with mycotoxins. The MOE is a risk assessment approach that indicates the level of health concern due to population exposure to compounds that are both genotoxic and carcinogenic (e.g., aflatoxins). Threshold doses are not set for compounds with both genotoxic and carcinogenic potentials due to potential health risks at low levels of exposure [59]; therefore, there are no tolerable daily intake (TDI) levels or health-based guidance values (HBGV) established for these compounds. Hence, there is a need for risk characterization using the MOE approach. Precisely, to estimate the MOE for AFB_1_ and total AF (sum of AFB_1_, AFB_2_, AFG_1_ and AFG_2_) in the population, the reference point benchmark dose lower confidence limit of 10% extra risk (BMDL_10_) value of 170 ng/kg bw/day for AFB_1_ from rodent data (EFSA, 2007) was divided by the respective APDI value (ng/kg bw/day) estimated in this study. The BMDL_10_ value of 170 ng/kg bw/day for AFB_1_ was applied in the MOE calculation for total AF on the conservative basis, as assumed by EFSA [63], that AFB_1_ constitutes most of the total aflatoxins in the food samples.

For other mycotoxins, the dietary exposure risks were characterized by comparing the estimated APDIs to their reference points or HBGV as stipulated by JECFA [75], EFSA [36], EFSA [73] and EFSA [74] for FUM, CIT, BEAU and MON, respectively. Accordingly, a group tolerable daily intake (TDI) of 2 μg/kg bw per day for sum of FB_1_, FB_2_ and FB_3_ was applied for FUM [75]. For CIT, the level of no concern for nephrotoxicity (0.2 μg CIT/kg bw per day) was adopted as appropriate to characterize the risk for nephrotoxicity due to the absence of any HBGV (e.g., TDI or tolerable weekly intake) resulting from substantial uncertainties in available toxicity data [36]. In accordance with EFSA [73] and EFSA [74], the lowest dose of 90 µg BEAU/kg bw per day and BMDL_05_ of 200 µg/kg bw per day were the reference points for calculating the MOEs for BEAU and MON, respectively, so that the risks would be indicative.

#### 4.4.3. Health Risk Assessment by Liver Cancer Burden Estimation

A further assessment of the health risks associated with dietary aflatoxin exposure among the consumers was performed by estimating the aflatoxin-induced primary liver cancer burden as the ingestion of AFB_1_ has been directly linked to hepatocellular carcinoma development in humans [60]. AFB_1_ was used for the estimation because its proportion outweighs the proportion of other aflatoxin types in food samples [63]. In addition, AFB_1_ synergistically interacts with hepatitis B virus (HBV) to increase the chances of hepatocellular carcinoma in humans by 30% [63]. Thus, the primary liver cancer cases (cancer/100,000 population/year) were estimated for the average food consumers by multiplying aflatoxin exposure (APDI) values by the average potency of HCC derived from the individual potencies of HBsAg^+^ (0.3) and HBsAg^-^ (0.01) groups [63] and respective chronic HBV infection rates in Nigeria (13.6 and 86.4% [61]). The formula is given as

Liver cancer rate = APDI × average potency

where average potency = (0.3 × 0.14) + (0.01 × 0.86) = 0.0506 cancers per year per 100,000 population per 1 ng/kg bw/day AFB_1_.

### 4.5. Data Analysis

All data analyses were performed using IBM SPSS v21.0 (SPSS^®^ Inc., Chicago, IL, USA). The distribution and occurrence levels of mycotoxins in the food types were analyzed by descriptive statistics, and the means of the mycotoxin concentrations (µg/kg) were compared. The unpaired student *t*-test (α = 0.05) was applied to compare the means of mycotoxin levels in the foods across the harvest and storage seasons (harvest and storage) and across the sampled states (Nasarawa and Niger).

In order to identify the factors determining awareness, the logit regression model according to Babalola et al. [93]) and Gujarati [115] was employed. This model is appropriate because the dependent variable was computed as a dummy variable (binary, scored as 0 for non-awareness of mycotoxin and 1 for awareness of mycotoxin). The model is specified as follows:

In (*P_i_*/(1 − *P_i_*)) = *β*_0_+ *β*_1_X_1_+ ……..+ *β*_n_X_n_ + *e_i_*

where *P_i_* = probability of respondents’ awareness of mycotoxin contamination in food; 1 − *P_i_* = probability of respondents’ non-awareness of mycotoxin; *β*_0_ = intercept; *β*_1_ (1, 2, 3, …, n) = regression coefficients; X_1_ (1, 2, 3, …, n) = independent variables; *e_i_* = error term.

## Figures and Tables

**Table 1:** Levels of major mycotoxins and their metabolites in grains consumed by households in north-central Nigeria.

Mycotoxins	Peanut (*n* ^a^ = 53)				Maize (*n* ^a^ = 142)				Rice (*n* ^a^ = 23)				Sorghum (*n* ^a^ = 24)			
		Concentration (µg/kg)				Concentration (µg/kg)				Concentration (µg/kg)				Concentration (µg/kg)		
	% ^b^	Range	Mean	Median	% ^b^	Range	Mean	Median	% ^b^	Range	Mean	Median	% ^b^	Range	Mean	Median
Aflatoxin B_1_ (AFB_1_)	64.2	0.36–6413	939	170	66.2	0.36–2277	99.4	10.1	56.5	0.36–416	44.0	1.61	45.8	0.36–138	19.4	3.11
AFB_2_	50.9	0.61–2007	289	53.0	42.3	0.11–189	14.6	2.23	26.1	0.22–25.3	5.18	0.65	29.2	0.11–20.3	3.87	1.55
AFG_1_	26.4	0.28–1355	210	5.22	33.1	0.27–1320	39.1	1.97	17.4	0.28–484	145	47.9	25.0	0.59–4.78	2.23	1.24
AFG_2_	11.3	2.76–519	151	68.2	7.7	0.85–77.1	10.9	2.77	8.7	6.31–30.2	18.3	18.3	0.0	<LOD	<LOD	<LOD
AFM_1_	41.5	0.79–534	80.8	29.2	25.4	0.22–123	10.4	1.72	8.7	3.35–25.0	14.2	14.2	16.7	0.22–4.54	1.60	0.81
AFM_2_	0.0	0.00	<LOD	<LOD	2.1	0.63–2.13	1.13	0.65	0.0	<LOD	<LOD	<LOD	0.0	<LOD	<LOD	<LOD
AFP_1_	0.0	0.00	<LOD	<LOD	2.8	0.96–13.2	7.52	7.96	0.0	<LOD	<LOD	<LOD	4.2	0.81	0.81	0.81
AFtot ^c^	64.2	0.28–8422	1281	249	66.9	0.36–3863	128	10.2	56.5	0.36–955	93.9	1.83	45.8	0.36–158	23.1	3.81
Beauvericin	47.2	0.02–3.48	0.65	0.37	63.4	0.05–385	8.14	0.87	52.2	0.05–2.37	0.56	0.43	70.8	0.07–4.75	1.15	0.64
Citrinin	3.8	5.09–14.5	9.82	9.82	57.0	5.64–51,195	2343	76.7	30.4	7.92–358	121	74.2	41.7	11.4–1335	186	29.6
Cyclopiazonic acid	13.2	67.8–6939	2345	785	12.7	7.08–6939	701	189	0.0	<LOD	<LOD	<LOD	0.0	<LOD	<LOD	<LOD
Dihydrocitrinone	0.0	<LOD	<LOD	<LOD	31.0	1.88–615	63.5	16.9	13.0	5.59–18.4	13.5	16.6	8.3	45.2–95.5	70.4	70.4
Fumonisin A_1_ (FA_1_)	0.0	<LOD	<LOD	<LOD	81.7	1.00–453	13.2	3.54	13.0	2.73–7.25	5.46	6.40	8.3	5.37–6.71	6.04	6.04
FA_2_	0.0	<LOD	<LOD	<LOD	63.4	3.99–1977	60.0	12.9	8.7	4.00	4.00	4.00	8.3	3.99–4.00	4.00	4.00
FB_1_	0.0	<LOD	<LOD	<LOD	93.0	22.9–47,168	2078	725	21.7	80.2–413	244	245	54.2	31.5–475	139	115
FB_2_	0.0	<LOD	<LOD	<LOD	90.1	23.0–13,013	558	194	26.1	9.96–107	54.3	47.3	50.0	3.50–111	36.7	33.7
FB_3_	0.0	<LOD	<LOD	<LOD	89.4	15.7–4949	239	102	21.7	15.7–58.8	34.2	38.7	41.7	8.25–54.0	22.4	19.0
FB_4_	0.0	<LOD	<LOD	<LOD	91.5	4.00–3073	141	57.2	21.7	8.65–28.7	19.9	22.9	41.7	4.00–35.1	16.0	19.4
FB_1_ + FB_2_^d^	0.0	<LOD	<LOD	<LOD	93.0	22.9–60,181	2619	928	26.1	9.96–520	257	226	54.2	35.0–585	173	139
ƩFB ^e^	0.0	<LOD	<LOD	<LOD	93.0	43.1–68,204	2988	1090	26.1	9.96–601	303	273	54.2	35.0–674	203	156
Hydrolysed FB_1_	0.0	<LOD	<LOD	<LOD	72.5	0.34–299	16.8	3.45	21.7	0.34–5.68	2.44	2.20	12.5	0.34–0.97	0.71	0.83
Moniliformin	3.8	2.50	2.50	2.50	64.1	2.50–990	72.1	23.5	30.4	2.50–37.0	11.1	6.47	91.7	2.50–199	60.9	32.7
Nivalenol	9.4	9.09–97.1	32.4	15.1	10.6	9.35–45.9	20.0	13.8	0.0	<LOD	<LOD	<LOD	0.0	<LOD	<LOD	<LOD
Ochratoxin A	3.8	0.65–1.34	1.00	1.00	3.5	0.76–93.6	26.2	5.13	4.3	59.0	59.0	59.0	12.5	3.64–10.1	6.72	6.45
Sterigmatocystin	39.6	0.13–30.1	6.43	3.71	26.1	0.13–11.8	1.44	0.37	47.8	0.13–2.25	0.96	0.82	12.5	0.13–2.47	0.96	0.29
Zearalenone	9.4	0.30–6.30	2.69	1.24	15.5	0.30–7.20	2.01	1.89	56.5	0.32–8.65	2.77	0.95	8.3	1.14–1.82	1.48	1.48

^a^ Number of samples analyzed. ^b^ Percentage of positive samples. ^c^ Summation of aflatoxin B_1_, aflatoxin B_2_, aflatoxin G_1_ and aflatoxin G_2_. ^d^ Summation of fumonisin B_1_ and fumonisin B_2_. ^e^ Summation of fumonisin B_1_, fumonisin B_2_, fumonisin B_3_ and fumonisin B_4_. Note: Positive samples/concentrations of trichothecenes were so low that they were not considered.

**Table 2:** Seasonal distribution of major mycotoxins in grains consumed by households in north-central Nigeria.

Season	Food	Percentage of Positive Samples (Mean Mycotoxin ^b^ Concentration (µg/kg))												
		AFB_1_	AFG_1_	AFtot	BEAU	CIT	CPA	DHC	FB_1_	ƩFB	MON	NIV	OTA	ZEN
Harvest (*n* ^a^ = 143) ^c^	Peanut (*n* ^a^ = 33)	76 (1110 *)	42 (210)	79 (1457 *)	21 (1.4)	6 (9.8)	18 (2724)	0 (<LOD)	0 (<LOD)	0 (<LOD)	6 (2.5)	9 (47.6)	6 (1.0)	15 (2.7)
	Maize (*n* ^a^ = 87)	69 (61)	26 (6.2)	70 (68.4)	53 (14.8)	46 (305)	9 (1398)	22 (21.4)	92 (2462 *)	92 (3554 *)	70 (63.2)	13 (20.0)	0 (<LOD)	17 (2.0)
	Rice (*n* ^a^ = 15)	67 (15.5)	20 (32.0)	67 (26.3)	33 (0.4)	27 (111)	0 (<LOD)	13 (12.0)	27 (243)	33 (289)	33 (6.3)	0 (<LOD)	0 (<LOD)	47 (2.2)
	Sorghum (*n* ^a^ = 5)	80 (42.0)	20 (1.2)	80 (48.1)	20 (2.0)	20 (1335)	0 (<LOD)	20 (95.5)	20 (475)	20 (675)	80 (9.0)	0 (<LOD)	20 (10.1)	0 (<LOD)
Storage (*n* ^a^ = 107) ^d^	Peanut (*n* ^a^ = 20)	45 (462)	0 (<LOD)	45 (630)	90 (0.4)	0 (<LOD)	5 (67.8)	0 (<LOD)	0 (<LOD)	0 (<LOD)	0 (<LOD)	10 (9.6)	0 (<LOD)	0 (<LOD)
	Maize (*n* ^a^ = 55)	62 (167 *)	44 (70.6 *)	62 (236 *)	80 (1.2)	75 (4331 *)	18 (144)	46 (95.5 *)	95 (1487)	95 (2117)	55 (90.1)	7 (19.9)	9 (26.2)	13 (2.0)
	Rice (*n* ^a^ = 8)	38 (139)	13 (484)	38 (319)	88 (0.7)	38 (133)	0 (<LOD)	13 (16.6)	13 (245)	13 (372)	25 (23.1)	0 (<LOD)	13 (59.0)	75 (3.5)
	Sorghum (*n* ^a^ = 19)	37 (6.5)	26 (2.4)	37 (8.8)	84 (1.1)	47 (58.7)	0 (<LOD)	5 (45.2)	63 (111)	63 (164)	95 (72.4)	0 (<LOD)	11 (5.0)	11 (1.5)

* Significant at *p* < 0.05 (student *t*-test) compared to the other season. ^a^ Number of samples analyzed. ^b^ AFB_1_: aflatoxin B_1_; AFtot: summation of aflatoxin B_1_, aflatoxin B_2_, aflatoxin G_1_ and aflatoxin G_2_; BEAU: beauvericin; CIT: citrinin; CPA: cyclopiazonic acid; DHC: dihydrocitrinone; FB_1_: fumonisin B_1_; ƩFB: summation of fumonisin B_1_, fumonisin B_2_, fumonisin B_3_ and fumonisin B_4_; MON: moniliformin; NIV: nivalenol; OTA: ochratoxin A; ZEN: zearalenone. ^c^ Total number of samples collected at harvest was 143, including three cowpea samples, one of which contained 122 µg/kg AFB_1_ and AF total, and 476 µg/kg CPA. ^d^ Total number of samples collected in storage was 107, including one millet sample, which contained only 49 µg/kg DON, and four cowpea samples, one of which contained 0.1 µg/kg BEAU.

**Table 3:** Occurrence of major mycotoxins in grains consumed by households in two north-central Nigerian states.

Mycotoxins	Nasarawa (*n* ^a^ = 170)				Niger (*n* ^a^ = 80)			
	% ^b^	Concentration (µg/kg)			% ^b^	Concentration (µg/kg)		
		Range	Mean	Median		Range	Mean	Median
Aflatoxin B_1_ (AFB_1_)	68.2	0.36–6413	326 *	15.6	46.3	0.36–2164	118	1.7
AFB_2_	51.8	0.11–2007	93.8 *	3.88	16.3	0.11–327	37.4	5.94
AFG_1_	37.1	0.27–1355	84.6 *	2.62	11.3	0.28–18.6	5.23	3.06
AFG_2_	10.6	0.85–519	58.9	6.52	1.3	1.74	1.74	1.74
AFM_1_	32.4	0.22–534	36.8	4	12.5	0.22–102	16.4	1.95
AFM_2_	1.8	0.63–2.13	1.13	0.65	0	<LOD	<LOD	<LOD
AFP_1_	2.9	0.81–13.2	6.18	5.42	0	<LOD	<LOD	<LOD
AFtot ^c^	68.2	0.28–8422	452 *	19.2	47.5	0.36–2510	129	1.72
Beauvericin	61.2	0.02–385	6.64	0.61	52.5	0.05–25.0	2.19	0.66
Citrinin	41.8	5.1–51195	2104 *	76.3	36.3	6.63–20290	1487	40.6
Cyclopiazonic acid	9.4	7.61–6939	1572 *	465	12.5	7.08–1175	436	392
Deoxynivalenol	0	<LOD	<LOD	<LOD	1.3	48.8	48.8	48.8
Dihydrocitrinone	22.9	1.88–615	58.4	17.5	12.5	1.88–330	69.9	23
Fumonisin A_1_ (FA_1_)	44.7	1.00–453	14.1	3.94	56.3	1.00–176	10.9	2.93
FA_2_	32.9	3.99–1977	66.3	15.3	47.5	3.99–389	44.7	10.3
FB_1_	53.5	31.5–47168	2041 *	681	73.8	22.9–18325	1552	645
FB_2_	52.4	3.50–13013	542 *	178	71.3	23.0–4380	421	156
FB_3_	50	8.25–4949	231 *	98.7	71.3	15.7–1494	195	89
FB_4_	51.2	4.00–3073	134 *	53.7	72.5	4.00–1036	119	46.3
FB_1_ + FB_2_^d^	54.1	9.96–60181	2543 *	841	73.8	22.9–22360	1959	804
ƩFB ^e^	54.1	9.96–68204	2883 *	985	73.8	80.3–24890	2264	932
Hydrolysed FB_1_	40	0.34–299	18.9	3.43	55	0.34–159	10.6	2.94
Moniliformin	48.2	2.50–574	73.8	34.8	51.3	2.50–990	53.9	8.79
Nivalenol	10	9.09–97.1	25	15.6	5	11.5–12.9	12.3	12.4
Ochratoxin A	6.5	0.65–93.6	19.3	5.13	0	<LOD	<LOD	<LOD
Zearalenone	18.2	0.30–8.65	2.7	1.87	13.8	0.30–3.59	1.18	0.32

* Significant at *p* < 0.05 (student t-test) compared to the other season. ^a^ Number of samples analyzed. ^b^ Percent positive samples. ^c^ Summation of aflatoxin B_1_, aflatoxin B_2_, aflatoxin G_1_ and aflatoxin G_2_. ^d^ Summation of fumonisin B_1_ and fumonisin B_2_. ^e^ Summation of fumonisin B_1_, fumonisin B_2_, fumonisin B_3_ and fumonisin B_4_.

**Table 4:** Percentage of foods in north-central Nigeria that present mycotoxin levels above stipulated maximum acceptable limits.

Foods	Percentage of Samples Exceeding Stipulated Limits				
	AFB_1_ (2 µg/kg) ^b^	AFtot (4 µg/kg) ^b^	AFtot (10 µg/kg) ^c^	FB_1_ + FB_2_ (1000 µg/kg) ^b^	OTA (5 µg/kg) ^b^
Cowpea (*n* ^a^ = 7)	14.2	14.2	14.2	-	-
Peanut (*n* ^a^ = 53)	56.6	50.9	50.9	-	-
Maize (*n* ^a^ = 142)	50.0	43.7	34.5	41.5	2.10
Rice (*n* ^a^ = 23)	26.1	21.7	13.0	-	4.30
Sorghum (*n* ^a^ = 24)	33.3	20.8	20.8	-	8.33
Millet (*n* ^a^ = 1)	-	-	-	-	-
All (*n* ^a^ = 250)	46.4	40.0	34.0	23.6	2.40

^a^ Number of samples analyzed. ^b^ European Union stipulated maximum acceptable limit for aflatoxin B_1_ (AFB_1_), total aflatoxins (summation of aflatoxin B_1_, aflatoxin B_2_, aflatoxin G_1_ and aflatoxin G_2_; AFtot), sum of fumonisin B_1_ and fumonisin B_2_. ^c^ Total aflatoxins (summation of aflatoxin B_1_, aflatoxin B_2_, aflatoxin G_1_ and aflatoxin G_2_; AFtot) limit in Nigeria.

**Table 5:** Mycotoxin exposure and liver cancer risk estimations in the average household population consumers of cereals and nuts in north-central Nigeria.

Food	Population	Average Probable Daily Intake (ng/kg bw/day or µg/kg bw/day) ^a^						Margin of Exposure ^b^		Liver Cancer Risk for AFB_1_ (Cancer/Year/ 100,000 Population) ^c^	Margin of Exposure ^d^		
		AFB_1_^e^	AFtot ^e^	BEAU ^e^	CIT ^e^	ƩFB ^e, g^	MON ^e^	AFB_1_^e^	AFtot ^e^		BEAU ^e^	CIT ^e^	MON ^e^
Peanut	Children	4883	6661	0.003	- ^f^	- ^f^	- ^f^	0.035	0.026	247	30,000	- ^f^	- ^f^
	Adolescents	1953	2664	0.001	- ^f^	- ^f^	- ^f^	0.087	0.064	99	90,000	- ^f^	- ^f^
	Adults	803	1096	0.001	- ^f^	- ^f^	- ^f^	0.21	0.16	41	90,000	- ^f^	- ^f^
Maize	Children	655	844	0.05	15.4	19.7	0.48	0.26	0.20	33	1800	0.013	417
	Adolescents	262	337	0.02	6.18	7.88	0.19	0.65	0.51	13	4500	0.03	1053
	Adults	108	139	0.009	2.54	3.24	0.08	1.58	1.23	6	10,000	0.08	2500
Rice	Children	194	414	0.003	0.53	1.34	0.05	0.88	0.41	10	30,000	0.4	4000
	Adolescents	77.6	166	0.001	0.21	0.54	0.20	2.19	1.03	4	90,000	1	1000
	Adults	31.9	68.1	0.0004	0.08	0.22	0.008	5.33	2.50	2	225,000	3	25,000
Sorghum	Children	56.8	67.7	0.003	0.55	0.60	0.18	2.99	2.51	3	30,000	0.4	1111
	Adolescents	22.7	27.1	0.001	0.22	0.24	0.07	7.48	6.28	1	90,000	1	2857
	Adults	9.35	11.1	0.001	0.09	0.10	0.03	18.2	15.3	1	90,000	2	6667

^a^ Average probable daily intake (APDI; ng/kg bw/day for aflatoxins and µg/kg bw/day for other mycotoxins) was estimated for the average population in north-central Nigeria according to EFSA [63] and JECFA [64] by multiplying mean mycotoxin concentration in food (ng/g) by average food consumption (g/person/d) data (groundnut: 52 [65]; maize: 65.9, rice: 44.1 and sorghum: 29.3 (based on consumption data in NBS/World Bank [66] LSMS—Panel (Wave 3), and Claro et al. [67] Adult Equivalent Conversion Factor based on WHO Recommended Dietary Allowances according to age and gender)); then, the product is divided by the average body weight of the population (assumed to be 10 kg for children and 25 kg for adolescents [68,69,70]; estimated as 60.8 kg for adults from questionnaire data in this study). ^b^ Margin of exposure estimation was determined according to EFSA [71] and JECFA [72] by dividing the benchmark-dose lower limit (BMDL10) value of 170 ng/kg bw/day established by the CONTAM Panel for AFB_1_ [63] with the APDI for AFB_1_ or total aflatoxins. ^c^ Primary liver cancer risk for AFB_1_ was estimated as new cancer cases/year/100,000 population (rounded to nearest whole number) by multiplying the AFB_1_ APDI by the average HCC potency (0.0506 based on 13.6% hepatitis B virus infection prevalence in Nigeria, [61]). ^d^ Margin of exposure (rounded to the nearest decimal or whole number) was estimated by dividing the reference point by the APDI. Reference point: beauvericin, at the lowest dose of 90 μg/kg bw per day [73]; moniliformin, BMDL_05_ of 200 μg/kg bw per day [74]; citrinin, a level of no concern for nephrotoxicity of 0.2 μg/kg bw per day [36]. ^e^ Mycotoxins: AFB_1_ (aflatoxin B_1_), AFtot (sum of aflatoxins B_1_, B_2_, G_1_ and G_2_), BEAU (beauvericin), CIT (citrinin), ƩFB (summation of fumonisins B_1_, B_2_, B_3_ and B_4_), MON (moniliformin). ^f^ APDI and MOE not estimated because all samples or just 4% (*n* = 2) samples contained mycotoxin. ^g^ Health based guidance value for fumonisin [75]: a group tolerable daily intake (TDI) of 2 μg/kg bw per day for sum of FB_1_, FB_2_, FB_3_ and FB_4_.

**Table 6:** Distribution of household farmers’ personal characteristics, grain handling and mycotoxin awareness variables.

Variables	Nasarawa	Niger	Variables	Nasarawa	Niger
***Age (years; mean ± SE) and Education:***	***n* = 82**	***n* = 73**	***Coping strategy with seed soiled by rain:***	***n* = 34**	***n* = 22**
Age	33.9 ±13.8	36.9 ± 16.6	Sun-drying	24 (70.6%)	20 (90.9%)
<Secondary	32 (39.0%)	33 (45.2%)	None	9 (26.5%)	2 (9.1%)
≥Secondary	50 (61.0%)	40 (54.8%)	***Grain processing method:***	***n* = 82**	***n* = 73**
***Sex:***	***n* = 82**	***n* = 73**	Sorting	74 (90.2%)	69 (94.5%)
Male	48 (58.5%)	35 (48.0%)	Winnowing	65 (79.3%)	46 (63.0%)
Female	34 (41.5%)	37 (50.7%)	Hot water washing	36 (43.9%)	15 (20.6%)
***Seed source:***	***n* = 82**	***n* = 73**	Cold water washing	28 (34.1%)	10 (13.7%)
Open market	5 (6.1%)	14 (19.2%)	Parboiling	10 (12.2%)	4 (5.5%)
Previous harvest	77 (93.9%)	59 (80.8%)	Fermenting	37 (45.1%)	17 (23.3%)
Seed type: Local/traditional	81 (98.8%)	69 (94.5%)	***Awareness of mycotoxins:***	***n* = 82**	***n* = 73**
***Pre-storage handling practice:***	***n* = 82**	***n* = 73**	Can identify mouldy food	69 (84.1%)	58 (79.5%)
Sun-drying	81 (98.8%)	65 (89.0%)	Have heard of mycotoxins	35 (42.7%)	11 (15.1%)
Shelling	21 (25.6%)	30 (41.1%)	Food handling practice to ameliorate mycotoxins	21 (25.6%)	8 (11.0%)
Packaging	59 (72.0%)	35 (48.0%)	***Handling practice to ameliorate mycotoxins:***	***n* = 21**	***n* = 8**
***Storage systems:***	***n* = 82**	***n* = 73**	Harvest grains early	2 (9.5%)	6 (75.0%)
None	2 (2.4%)	11 (15.1%)	Use of chemicals	4 (19.1%)	2 (25.0%)
Bags	74 (90.2%)	54 (74.0%)	Smoking/drying	10 (47.6%)	-
Plastic container	2 (2.4%)	2 (2.7%)	Adequate storage	5 (23.8%)	-
***Rhumbu***	2 (2.4%)	4(5.5%)	***Source of awareness:***	***n* = 35**	***n* = 11**
Kitchen roof	2 (2.4%)	2 (2.7%)	Mass media	9 (25.7%)	2 (18.2%)
Protection of stored grains from rain & water	48 (58.5%)	51 (69.9%)	Community campaign	16 (45.7%)	5 (45.5%)
***Storage period:***	***n* = 82**	***n* = 73**	Extension education	10 (28.6%)	4 (36.4%)
<1 month	-	5 (6.9%)			
1–3 months	19 (23.2%)	10 (13.7%)			
>3 month	63 (76.8%)	57 (78.1%)			

**Table 7:** Logit regression result showing factors influencing awareness of mycotoxins among respondents in the selected study areas.

Independent Variables	Beta Coefficient		
	Nasarawa	Niger	Pooled
Constant	−2.844 (1.311) *	−22.175 (11896.4) **	−3.57 (1.14) **
Sex (male = 1; female = 0)	0.816 (0.628)	−0.206 (1.146)	0.541 (0.016)
Age (years)	0.017 (0.023)	0.019 (0.032)	0.008 (0.016)
Education (<2° = 0; ≥2° =1)	1.271 (0.643) *	0.311 (0.108) **	0.156 (0.074) *
Consumption of spoilt food (yes = 1; no = 0)	0.879 (0.773)	1.727 (1.168)	0.841 (0.530)
Ability to identify mouldy grains (yes = 1; no = 0)	1.952 (1.166)	8.016 (7.123)	1.689 (1.064)
Exposure to food handling practice for ameliorating food pathogens (yes = 1; no = 0)	2.137 (0.703) **	1.725 (0.788) *	2.315 (0.562) **
Existence of food related sickness in the family (yes = 1; no = 0)	−0.489 (−0.489)	0.392 (1.255)	0.092 (0.081)
Chi-square	25.01 **	93.00 **	61.53 **
Nagelkerke R Square	0.508	0.533	0.515
Cox and Snell R Square	0.456	0.428	0.337
−2 Log likelihood	185.783	322.494	116.35

* Significant at 5%. ** Significant at 1%. Standard Error in parenthesis.

## Data Availability

The data presented in this study are available in Supplementary Materials.

## Supplementary Materials

The following are available online at https://www.mdpi.com/article/10.3390/toxins13090635/s1, Table S1: Occurrence of microbial and plant metabolites cowpea and peanuts consumed by households in north-central Nigeria, Table S2: Occurrence of microbial and plant metabolites maize, rice and sorghum consumed by households in north-central Nigeria.
