# Supplementary material for: Dietary Risk Assessment and Consumer Awareness of Mycotoxins among Household Consumers of Cereals, Nuts and Legumes in North-Central Nigeria
Source: Toxins (Basel). 2021 Sep 9;13(9):635. doi: 10.3390/toxins13090635 (PMC8472633; doi:10.3390/toxins13090635)
Supplement: Supplementary file 1 [file toxins-13-00635-s001.zip › toxins-1339067-su-9.9-9.16.pdf]

Dietary Risk Assessment and Consumer Awareness of Mycotoxins Among Household Consumers of Cereals, Nuts and Legumes in North-central Nigeria

Chibundu N. Ezekiel, Kolawole I. Ayeni, Muiz O. Akinyemi, Michael Sulyok, Oluwawapelumi A. Oyedele, Daniel A. Babalola, Isaac M. Ogara and Rudolf Krska

Table S1. Occurrence of microbial and plant metabolites cowpea and peanuts consumed by households in north-central Nigeria.

	Cowpea (n[a]=7)				Groundnut (n[a]=53)			
		Concentration (µg/kg)				Concentration (µg/kg)		
Metabolites	%[b]	Range	Mean	Median	%[b]	Range	Mean	Median
3-Nitropropionic acid	14.3	4.2	4.2	4.2	47.2	4.69-334	66.3	28.7
7-Hydroxykaurenolide	42.9	1.47-10.6	5.4	4.2	45.3	3.46-96.9	25.0	21.2
7-Hydroxypestalotin	0.0	<LOD	<LOD	<LOD	7.6	8.77-23.3	13.5	11.0
Aflatoxicol	0.0	<LOD	<LOD	<LOD	37.7	1.42-878	127	22.7
Alternariolmethylether	0.0	<LOD	<LOD	<LOD	3.8	0.3	0.3	0.3
Andrastin A	0.0	<LOD	<LOD	<LOD	45.3	0.88-45.6	5.1	2.4
Anisomycin	14.3	2.2	2.2	2.2	5.7	12.8-20.3	15.6	13.8
Ascochlorin	0.0	<LOD	<LOD	<LOD	1.9	0.1	0.1	0.1
Asperfuran	14.3	376[c]	376[c]	376[c]	32.1	27.2-149040[c]	9689[c]	62.7[c]
Asperglaucide	100	2.60-22.6	11.4	10.3	86.8	0.11-4092	333	52.9
Asperphenamate	85.7	0.50-31.0	8.4	4.2	86.8	0.50-345	28.8	4.0
Aurofusarin	0.0	<LOD	<LOD	<LOD	11.3	5.0-38.0	20.4	17.7
Averantin	14.3	3.5	3.5	3.5	52.8	0.45-249	40.2	12.7
Averufin	14.3	10.4	10.4	10.4	66.0	0.32-272	53.7	17.2
Bikaverin	0.0	<LOD	<LOD	<LOD	28.3	4.51-163	33.9	13.8
Chloramphenicol	14.3	0.2	0.2	0.2	0.0	<LOD	<LOD	<LOD
Chrysogin	0.0	<LOD	<LOD	<LOD	24.5	0.95-8.53	2.9	1.0

Citreohybridinol	0.0	<LOD	<LOD	<LOD	1.9	9.2	9.2	9.2
Citreorosein	0.0	<LOD	<LOD	<LOD	20.8	1.24-8.41	4.0	4.1
Curvularin	0.0	<LOD	<LOD	<LOD	1.9	4.5	4.5	4.5
cyclo(L-Pro-L-Tyr)	100	14.3-134	48.6	37.8	69.8	1.9-277	23.6	12.8
cyclo(L-Pro-L-Val)	57.1	4.74-25.3	12.0	8.9	32.1	1.90-37.2	10.9	8.3
Cytochalasin H	0.0	<LOD	<LOD	<LOD	5.7	57.5-230	117	63.6
Cytochalasin J	0.0	<LOD	<LOD	<LOD	5.7	11.5-383	149	52.1
Daidzein	85.7	61.8-2044	813	630	64.2	1.50-105	10.9	5.2
Daidzin	85.7	90.8-1377	469	344	3.8	1.50-27.4	14.5	14.5

Table S1. Continued.

	Cowpea (n[a]=7)				Groundnut (n[a]=53)			
		Concentration (μg/kg)				Concentration (μg/kg)		
Metabolites	%[b]	Range	Mean	Median	%[b]	Range	Mean	Median
Diacetoxyscirpenol	0.0	<LOD	<LOD	<LOD	22.6	0.25-8.21	1.8	0.9
Emodin	28.6	0.18-1.25	0.7	0.7	62.3	0.18-8.45	1.8	0.7
Endocrocin	0.0	<LOD	<LOD	<LOD	3.8	25.6-108	66.8	66.8
Epiequisetin	0.0	<LOD	<LOD	<LOD	88.7	0.16-111	10.4	4.2
Equisetin	28.6	1.15-18.5	9.8	9.8	96.2	1.19-905	181	103.7
Fallacinol	0.0	<LOD	<LOD	<LOD	3.8	1.83-12.7	7.2	7.2
Fellutanine A	0.0	<LOD	<LOD	<LOD	1.9	4.6	4.6	4.6
Flavoglaucin	28.6	1.45-2.05	1.7	1.7	50.9	0.99-1718	126	13.5
Fusapyron	0.0	<LOD	<LOD	<LOD	3.8	1.22-5.43	3.3	3.3
Fusaric acid	0.0	<LOD	<LOD	<LOD	1.9	440	440	440
Fusarinolic acid	14.3	36.9	36.9	36.9	24.5	6.50-953	141	21.3
Genistein	57.1	273-1675	881	787	24.5	5.61-48.6	11.8	9.4
Genistin	57.1	170-697	310	186	0.0	<LOD	<LOD	<LOD
Iso-Rhodoptilometrin	0.0	<LOD	<LOD	<LOD	30.2	0.06-1.53	0.5	0.3
Kojic acid	100	34.0-964	200	82.3	49.1	33.5-92292	8594	1155
Lecanoic acid	0.0	<LOD	<LOD	<LOD	3.8	1.85-3.43	2.6	2.6

Macrosporin	14.3	2.8	2.8	2.8	11.3	0.22-0.74	0.5	0.5
Methylfunicone	0.0	<LOD	<LOD	<LOD	1.9	2.4	2.4	2.4
Monocerin	0.0	<LOD	<LOD	<LOD	7.5	0.42-4.71	2.0	1.3
N-Benzoyl-Phenylalanine	28.6	1.77-45.0	23.4	23.4	41.5	0.25-186	20.6	2.7
Neoechinulin A	0.0	<LOD	<LOD	<LOD	7.5	14.8-735	241	107.9
Neoechinulin D	0.0	<LOD	<LOD	<LOD	7.5	3.09-130	52.0	37.3
Nidurufin	0.0	<LOD	<LOD	<LOD	9.4	1.71-20.0	5.8	2.4
Nigragillin	42.9	31152-80640[c]	52096[c]	44496[c]	22.6	14352-5186400[c]	875064[c]	66436[c]
Norsolorinic acid	14.3	1.9	1.9	1.9	52.8	1.61-114	26.3	9.8
O-Methylsterigmatocystin	14.3	0.8	0.8	0.8	54.7	0.12-345	30.2	6.0

Table S1. Continued.

	Cowpea (n[a]=7)				Groundnut (n[a]=53)			
		Concentration (µg/kg)				Concentration (µg/kg)		
Metabolites	%[b]	Range	Mean	Median	%[b]	Range	Mean	Median
Pestalotin	0.0	<LOD	<LOD	<LOD	7.5	4.52-10.6	7.0	6.4
Phenopyrrozin	57.1	3.59-59.1	28.3	25.2	7.5	2.57-44.7	14.4	5.2
Physcion	0.0	<LOD	<LOD	<LOD	3.8	99.4-169	134	134
Purpactin A	14.3	1.4	1.4	1.4	13.2	0.59-1.96	0.9	0.6
Questiomycin A	0.0	<LOD	<LOD	<LOD	9.4	1.35-7.98	4.8	5.6
Quinolactacin A	14.3	6.8	6.8	6.8	52.8	0.02-63.0	5.3	0.4
Quinolactacin B	14.3	0.0	0.0	0.0	15.1	0.02-1.66	0.5	0.3
Siccanol	0.0	<LOD	<LOD	<LOD	1.9	362	362	362
Skyrin	0.0	<LOD	<LOD	<LOD	3.8	0.64-1.75	1.2	1.2
Sydowinin A	42.9	4.82-12.2	7.3	4.8	15.1	4.82-14.1	7.4	4.8
Tenuazonic acid	0.0	<LOD	<LOD	<LOD	1.9	366	366	366
Tryptophol	100	40.6-267	89.8	67.9	96.2	6.94-595	48.4	36.8
Verruculotoxin	0.0	<LOD	<LOD	<LOD	15.1	0.30-4.91	2.2	1.6
Versicolorin A	14.3	3.1	3.1	3.1	50.9	0.62-47.4	11.0	3.0
Versicolorin C	14.3	2.4	2.4	2.4	54.7	0.25-265	47.6	22.1

Versiconal Acetate	0.0	<LOD	<LOD	<LOD	1.9	2.7	2.7	2.7
W494	0.0	<LOD	<LOD	<LOD	9.4	1.09-26.4	9.8	6.8

[a]Number of samples analyzed. [b]Percent positive samples. [c]Values represent peak areas due to lack of standard.

Table S2. Occurrence of microbial and plant metabolites maize, rice and sorghum consumed by households in north-central Nigeria.

	Maize (n[a]=142)				Rice (n[a]=23)				Sorghum (n[a]=24)			
		Concentration (μg/kg)				Concentration (μg/kg)				Concentration (μg/kg)		
Metabolites	%[b]	Range	Mean	Median	%[b]	Range	Mean	Median	%[b]	Range	Mean	Median
3-Nitropropionic acid	71.8	1.23-6243	181	43.2	52.2	1.23-94.7	20.0	12.0	62.5	2.55-99.3	22.5	8.4
7-Hydroxykaurenolide	2.8	1.47-20.0	10.8	10.9	26.1	1.47-5.44	3.8	4.5	8.3	20.5-52.9	36.7	36.7
7-Hydroxypestalotin	87.3	0.82-106	14.7	7.7	17.4	4.48-32.1	14.8	11.3	25.0	2.04-44.1	13.6	5.0
Aflatoxicol	16.2	0.38-177	14.3	2.4	0.0	<LOD	<LOD	<LOD	4.2	4.0	4.0	4.0
Agroclavine	5.6	0.16-0.94	0.4	0.3	4.3	1.08	1.1	1.1	37.5	0.16-8.06	1.7	0.4
Alternariolmethylether	5.6	0.25-9.89	1.7	0.4	4.3	0.25	0.3	0.3	0.0	<LOD	<LOD	<LOD
Altersolanol	2.8	43.6-169	80.2	54.3	0.0	<LOD	<LOD	<LOD	37.5	58.0-1391	488	343
Andrastin A	3.5	0.93-11.5	4.3	1.4	8.7	1.06-4.76	2.9	2.9	12.5	0.40-1.85	1.2	1.5
Anisomycin	5.6	2.15-25.3	12.3	10.4	0.0	<LOD	<LOD	<LOD	0.0	<LOD	<LOD	<LOD
Antibiotic F 1849 A	1.4	21.6-25.8	23.7	23.7	0.0	<LOD	<LOD	<LOD	0.0	<LOD	<LOD	<LOD
Ascochlorin	4.9	0.27-72.5	11.0	0.7	0.0	<LOD	<LOD	<LOD	0.0	<LOD	<LOD	<LOD
Ascofuranone	0.7	3.4	3.4	3.4	0.0	<LOD	<LOD	<LOD	0.0	<LOD	<LOD	<LOD
Asperfuran	16.2	10.7-484320[c]	76098[c]	126[c]	17.4	42.6-565[c]	219[c]	135[c]	4.2	30.9[c]	30.9[c]	30.9[c]
Asperglaucide	92.3	0.11-1547	173	32.7	87.0	2.54-1152	157	18.1	95.8	1.07-1977	148	27.3
Asperphenamate	89.4	0.5-2243	61.5	4.9	91.3	0.50-384	44.5	6.5	91.7	1.34-958	86.9	7.6
Aurofusarin	2.8	5.0-15.9	9.5	8.5	0.0	<LOD	<LOD	<LOD	4.2	14.1	14.1	14.1
Averantin	32.4	0.26-42.8	4.3	1.1	13.0	2.74-4.29	3.4	3.3	16.7	0.38-7.00	2.3	1.0
Averufin	57.0	0.20-291	8.5	1.0	39.1	0.17-12.3	3.4	0.5	45.8	0.18-22.4	3.0	0.8
Bafilomycin A1	1.4	86.5-98.8	92.7	92.7	0.0	<LOD	<LOD	<LOD	0.0	<LOD	<LOD	<LOD
Bikaverin	76.8	5.0-979	53.2	19.2	26.1	5.00-54.2	15.8	7.9	83.3	5.00-204	40.5	21.8
Butenolid	6.3	17.2-175	39.1	20.4	0.0	<LOD	<LOD	<LOD	0.0	<LOD	<LOD	<LOD
Cercosporin	0.0	<LOD	<LOD	<LOD	0.0	<LOD	<LOD	<LOD	8.3	24.6-24.6	24.6	24.6
Chanoclavin	26.8	0.03-3.88	0.5	0.1	4.3	0.30	0.3	0.3	8.3	0.30-2.09	1.2	1.2
Chevalone B	7.7	0.42-1.19	0.5	0.4	0.0	<LOD	<LOD	<LOD	0.0	<LOD	<LOD	<LOD
Chloramphenicol	0.7	0.2	0.2	0.2	4.3	0.19	0.2	0.2	0.0	<LOD	<LOD	<LOD

Chlorocitreorosein	1.4	30.0-151	90.6	90.6	0.0	<LOD	<LOD	<LOD	4.2	1.4	1.4	1.4

Table S2. Continued.

	Maize (n[a]=142)				Rice (n[a]=23)				Sorghum (n[a]=24)			
		Concentration (μg/kg)				Concentration (μg/kg)				Concentration (μg/kg)		
Metabolites	%[b]	Range	Mean	Median	%[b]	Range	Mean	Median	%[b]	Range	Mean	Median
Chrysogin	7.0	0.95-32.3	6.9	4.0	8.7	0.95-0.95	1.0	1.0	0.0	<LOD	<LOD	<LOD
Citreohybridinol	1.4	3.27-423	213	213	0.0	<LOD	<LOD	<LOD	0.0	<LOD	<LOD	<LOD
Citreorosein	28.9	1.24-74.2	8.1	4.0	30.4	1.24-3.87	1.6	1.3	16.7	1.24-5.90	3.2	2.9
Citreoviridin	1.4	11.1-499	255	255	0.0	<LOD	<LOD	<LOD	0.0	<LOD	<LOD	<LOD
Citromycetin	0.7	13.0	13.0	13.0	4.3	5.00	5.0	5.0	0.0	<LOD	<LOD	<LOD
Curvularin	3.5	2.51-7.89	5.3	5.5	4.3	1.00	1.0	1.0	8.3	9.55-12.5	11.0	11.0
cyclo(L-Pro-L-Tyr)	97.9	1.90-253	21.4	12.5	91.3	1.90-115	40.9	29.5	91.7	5.74-229	32.0	15.5
cyclo(L-Pro-L-Val)	83.1	1.9-205	18.0	10.1	87.0	1.90-48.7	19.7	19.1	25.0	1.90-32.6	13.2	11.3
Cylindrol B	1.4	0.05-3.18	1.6	1.6	0.0	<LOD	<LOD	<LOD	0.0	<LOD	<LOD	<LOD
Daidzein	15.5	1.50-799	122	13.8	43.5	1.50-301	45.8	8.2	8.3	7.91-48.3	28.1	28.1
Daidzin	20.4	1.50-38904	1377	8.2	13.0	7.61-15.9	11.9	12.0	29.2	10.3-28.7	17.5	16.2
Deoxyfusapyron	15.5	2.75-105	18.4	9.3	0.0	<LOD	<LOD	<LOD	0.0	<LOD	<LOD	<LOD
Diacetoxyscirpenol	49.3	0.25-57.1	6.3	2.4	34.8	1.24-12.0	3.7	2.2	37.5	0.66-10.6	2.5	1.2
Dichlordiaportin	4.9	3.34-69.2	20.3	14.8	0.0	<LOD	<LOD	<LOD	37.5	6.99-56.8	24.5	18.8
Diplodiatoxin	4.2	14.0	71.0	74.4	0.0	<LOD	<LOD	<LOD	0.0	<LOD	<LOD	<LOD
Elymoclavine	2.8	0.43-2.23	1.4	1.4	4.3	2.23	2.2	2.2	33.3	0.43-13.2	2.8	1.0
Emodin	69.7	0.18-53.5	1.7	0.5	78.3	0.18-2.11	0.8	0.6	87.5	0.18-10.4	1.6	1.0
Endocrocin	7.7	25.6-275	94.1	65.6	0.0	<LOD	<LOD	<LOD	4.2	25.6	25.6	25.6
Epiequisetin	83.8	0.16-60.5	5.1	1.4	95.7	0.16-27.5	5.5	2.1	50.0	0.16-7.00	1.5	0.5
Equisetin	93.7	0.74-1116	47.3	10.3	95.7	1.15-233	44.0	12.3	91.7	1.15-339	32.3	10.5
Fallacinol	19.0	0.18-31.3	4.3	1.6	8.7	0.44-2.82	1.6	1.6	8.3	4.41-4.85	4.6	4.6
Fellutanine A	4.2	12.8	7.1	6.6	4.3	1.65	1.7	1.7	0.0	<LOD	<LOD	<LOD
Festuclavine	0.7	0.03	0.0	0.0	8.7	0.07-0.27	0.2	0.2	4.2	0.2	0.2	0.2
Flavoglaucin	81.0	0.17-3825	105	5.6	73.9	0.14-4871	295	7.4	62.5	0.50-1221	138	8.9

Fusaproliferin	3.5	38.1-363	161	157	0.0	<LOD	<LOD	<LOD	0.0	<LOD	<LOD	<LOD
Fusapyron	4.9	2.49-24.9	12.7	10.8	8.7	1.22-8.12	4.7	4.7	0.0	<LOD	<LOD	<LOD

Table S2. Continued.

	Maize (n[a]=142)				Rice (n[a]=23)				Sorghum (n[a]=24)			
		Concentration (µg/kg)				Concentration (µg/kg)				Concentration (µg/kg)		
Metabolites	%[b]	Range	Mean	Median	%[b]	Range	Mean	Median	%[b]	Range	Mean	Median
Fusaric acid	23.9	39.5-57688	2092	213	0.0	<LOD	<LOD	<LOD	4.2	416	416	416
Fusarinolic acid	54.9	6.50-10327	834	327	17.4	6.50-223	160	205	12.5	43.2-76.2	59.0	57.7
Genistein	6.3	1.06-494	61.2	5.9	17.4	4.33-30.1	14.1	11.0	12.5	7.98-13.3	9.9	8.4
Genistin	14.8	2.66-448	34.9	12.6	4.3	6.78	6.8	6.8	20.8	10.1-23.4	17.7	18.4
Griseofulvin	0.0	<LOD	<LOD	<LOD	0.0	<LOD	<LOD	<LOD	8.3	0.23-2.65	1.4	1.4
Ilicicolin B	4.9	0.97-15.1	5.4	2.4	17.4	0.97-10.1	3.7	1.9	0.0	<LOD	<LOD	<LOD
Ilicicolin E	2.8	0.05-590	1.6	0.2	0.0	<LOD	<LOD	<LOD	0.0	<LOD	<LOD	<LOD
Ilicicolin F	2.1	0.76-43.7	15.3	1.5	0.0	<LOD	<LOD	<LOD	0.0	<LOD	<LOD	<LOD
Infectopyron	0.0	<LOD	<LOD	<LOD	8.7	17.4-23.4	20.4	20.4	0.0	<LOD	<LOD	<LOD
Iso-Rhodoptilometrin	64.1	0.03-5.45	0.7	0.3	52.2	0.06-0.50	0.2	0.2	54.2	0.08-1.51	0.4	0.2
Kojic acid	80.3	25.6-125187	4021	807	73.9	15.3-59200	4200	270	58.3	29.1-3696	629	227
Kotanin A	0.0	<LOD	<LOD	<LOD	4.3	3.94	3.9	3.9	0.0	<LOD	<LOD	<LOD
Lecanoic acid	5.6	1.22-14.1	5.7	3.3	4.3	4.02	4.0	4.0	8.3	1.13-1.47	1.3	1.3
Linamarin	17.6	39.8-199111	18558	296	0.0	<LOD	<LOD	<LOD	25.0	40.7-3145	870	199
LL-Z 1272e	0.7	1.82	1.8	1.8	0.0	<LOD	<LOD	<LOD	0.0	<LOD	<LOD	<LOD
Lotaustralin	15.5	9.10-12446	1198	41.7	0.0	<LOD	<LOD	<LOD	20.8	12.0-198	68.0	20.8
Macrosporin	44.4	0.22-23.0	1.2	0.5	60.9	0.22-14.9	2.6	0.4	66.7	0.43-31.9	5.6	2.2
Malformin C	1.4	2.17-26.1	14.1	14.1	0.0	<LOD	<LOD	<LOD	0.0	<LOD	<LOD	<LOD
Methylfunicone	2.1	0.55-21.7	7.7	0.7	4.3	38.1	38.1	38.1	0.0	<LOD	<LOD	<LOD
Monactin	3.5	0.31-359	133	7.9	0.0	<LOD	<LOD	<LOD	0.0	<LOD	<LOD	<LOD
Monoacetoxyscirpenol	12.7	2.60-55.0	15.2	6.4	0.0	<LOD	<LOD	<LOD	4.2	7.4	7.4	7.4
Monocerin	61.3	0.20-119	9.3	1.6	56.5	0.10-6.08	1.6	1.2	58.3	0.22-13.4	4.0	2.4
N-Benzoyl-Phenylalanine	65.5	0.25-771	21.2	2.1	82.6	0.50-212	24.4	2.9	54.2	0.25-287	46.8	2.4

Neoechinulin A	12.0	6.85-2468	308	93.2	17.4	2.26-33.1	16.5	15.3	16.7	2.26-230	64.5	13.1
Neoechinulin D	8.5	2.12-359	63.4	18.2	8.7	1.64-3.31	2.5	2.5	4.2	47.7	47.7	47.7
Nidurufin	6.3	0.13-16.6	2.5	0.6	4.3	2.41	2.4	2.4	0.0	<LOD	<LOD	<LOD

Table S2. Continued.

	Maize (n[a]=142)				Rice (n[a]=23)				Sorghum (n[a]=24)			
		Concentration (μg/kg)				Concentration (μg/kg)				Concentration (μg/kg)		
Metabolites	%[b]	Range	Mean	Median	%[b]	Range	Mean	Median	%[b]	Range	Mean	Median
Nigragillin	35.2	10656-13296000[c]	426598[c]	44532[c]	26.1	21376-11424000[c]	1951560[c]	60232[c]	20.8	25416-104400[c]	49571[c]	43976[c]
Nonactin	2.1	1.90-101	64.9	92.4	0.0	<LOD	<LOD	<LOD	0.0	<LOD	<LOD	<LOD
Norsolorinic acid	26.8	1.07-101	12.2	3.1	13.0	2.12-8.23	5.9	7.2	12.5	0.88-3.90	2.2	1.9
O-Methylsterigmatocystin	33.8	0.12-127	4.9	1.0	17.4	0.12-12.1	7.2	8.3	16.7	0.24-6.10	1.8	0.5
Oxaline	6.3	0.13-0.71	0.3	0.1	0.0	<LOD	<LOD	<LOD	0.0	<LOD	<LOD	<LOD
Pestalotin	81.7	1.50-172	11.9	5.0	17.4	1.50-8.53	5.1	5.1	25.0	1.97-10.2	5.1	3.3
Phenopyrrozin	0.7	0.6	0.6	0.6	0.0	<LOD	<LOD	<LOD	4.2	1.4	1.4	1.4
Physcion	5.6	22.9-365	84.9	46.9	0.0	<LOD	<LOD	<LOD	0.0	<LOD	<LOD	<LOD
Pinselin	7.0	1.10-13.2	6.3	5.5	4.3	10.59	10.6	10.6	0.0	<LOD	<LOD	<LOD
Purpactin A	1.4	0.59-0.59	0.6	0.6	0.0	<LOD	<LOD	<LOD	0.0	<LOD	<LOD	<LOD
Pyrophen	0.0	<LOD	<LOD	<LOD	8.7	1.60-4.15	2.9	2.9	0.0	<LOD	<LOD	<LOD
Questiomycin A	92.3	3.81-408	30.7	15.8	21.7	6.63-19.5	11.1	9.1	37.5	1.37-17.8	6.4	4.9
Quinolactacin A	85.9	0.02-316	12.3	1.5	73.9	0.02-21.6	2.1	0.4	41.7	0.34-11.9	2.8	0.8
Quinolactacin B	51.4	0.02-8.93	1.0	0.2	34.8	0.02-0.63	0.2	0.1	33.3	0.05-0.60	0.2	0.1
Radicicol	13.4	0.85-64.1	14.0	8.1	0.0	<LOD	<LOD	<LOD	4.2	4.0	4.0	4.0
Roridin A	1.4	1.70-5.76	3.7	3.7	0.0	<LOD	<LOD	<LOD	0.0	<LOD	<LOD	<LOD
Rugulovasine A	34.5	12.7-781	56.7	12.7	0.0	<LOD	<LOD	<LOD	0.0	<LOD	<LOD	<LOD
Scalusamid A	9.2	0.53-15.8	3.0	1.6	0.0	<LOD	<LOD	<LOD	8.3	1.16-1.52	1.3	1.3
Sclerotin A	5.6	61.0-148	91.1	86.2	0.0	<LOD	<LOD	<LOD	0.0	<LOD	<LOD	<LOD
Secalonic acid D	1.4	33.3-148	90.5	90.5	0.0	<LOD	<LOD	<LOD	0.0	<LOD	<LOD	<LOD
Siccanol	14.1	134-9520	1016	328	4.3	469	469	469	0.0	<LOD	<LOD	<LOD
Skyrin	18.3	0.74-78.1	10.1	3.5	4.3	3.68	3.7	3.7	4.2	1.2	1.2	1.2

Stachybotryamide	1.4	60.1-60.5	60.3	60.3	0.0	<LOD	<LOD	<LOD	0.0	<LOD	<LOD	<LOD
Stachybotrylactam	1.4	9.00-9.69	9.3	9.3	0.0	<LOD	<LOD	<LOD	0.0	<LOD	<LOD	<LOD
Surfactin A	0.7	86720[c]	86720[c]	86720[c]	8.7	86000-137040[c]	111520[c]	111520[c]	0.0	<LOD	<LOD	<LOD

Table S2. Continued.

	Maize (n[a]=142)				Rice (n[a]=23)				Sorghum (n[a]=24)			
		Concentration (µg/kg)				Concentration (µg/kg)				Concentration (µg/kg)		
Metabolites	%[b]	Range	Mean	Median	%[b]	Range	Mean	Median	%[b]	Range	Mean	Median
Surfactin B	2.8	21512-83520[c]	43582[c]	34648[c]	8.7	32712-100560[c]	66636[c]	66636[c]	0.0	<LOD	<LOD	<LOD
Sydowinin A	0.7	4.82	4.8	4.8	0.0	<LOD	<LOD	<LOD	0.0	<LOD	<LOD	<LOD
Tentoxin	0.7	0.55	0.55	0.55	0.0	<LOD	<LOD	<LOD	0.0	<LOD	<LOD	<LOD
Tenuazonic acid	0.0	<LOD	<LOD	<LOD	30.4	15.0-75.2	46.7	41.1	0.0	<LOD	<LOD	<LOD
Trichodermin	2.1	11.6-45.3	27.0	24.1	0.0	<LOD	<LOD	<LOD	0.0	<LOD	<LOD	<LOD
Tryptophol	54.2	1.50-2010	121	30.4	78.3	6.43-97.2	37.9	36.0	37.5	12.2-91.9	39.7	18.4
Usnic acid	1.4	0.22-0.34	0.3	0.3	0.0	<LOD	<LOD	<LOD	4.2	0.3	0.2	0.2
Valinomycin	1.4	179-211	195	195	0.0	<LOD	<LOD	<LOD	0.0	<LOD	<LOD	<LOD
Versicolorin A	25.4	0.25-35.1	4.2	0.9	13.0	3.33-7.45	4.9	3.9	12.5	0.45-8.38	3.2	0.8
Versicolorin C	42.3	0.25-194	10.5	2.1	30.4	0.25-27.6	7.6	0.9	20.8	1.53-22.3	8.1	2.3
Versiconal Acetate	0.7	6.03	6.0	6.0	0.0	<LOD	<LOD	<LOD	0.0	<LOD	<LOD	<LOD

[a]Number of samples analyzed. [b]Percent positive samples. [c]Values represent peak areas due to lack of standard.
